# Peptide-Based Selective Inhibitors of Matrix Metalloproteinase-Mediated Activities

**DOI:** 10.3390/molecules171214230

**Published:** 2012-11-30

**Authors:** Margaret W. Ndinguri, Manishabrata Bhowmick, Dorota Tokmina-Roszyk, Trista K. Robichaud, Gregg B. Fields

**Affiliations:** 1 Torrey Pines Institute for Molecular Studies, 11350 SW Village Parkway, Port St. Lucie, FL 34987, USA; 2 Department of Chemistry, Eastern Kentucky University, 521 Lancaster Avenue, Richmond, KY 40475, USA; 3 Department of Periodontics, University of Texas Health Science Center, 7703 Floyd Curl Drive, San Antonio, TX 78229, USA; 4 Department of Biochemistry, University of Texas Health Science Center, 7703 Floyd Curl Drive, San Antonio, TX 78229, USA

**Keywords:** matrix metalloproteinase, protease inhibitor, secondary binding site, cyclic peptide, phage display, triple-helix

## Abstract

The matrix metalloproteinases (MMPs) exhibit a broad array of activities, some catalytic and some non-catalytic in nature. An overall lack of selectivity has rendered small molecule, active site targeted MMP inhibitors problematic in execution. Inhibitors that favor few or individual members of the MMP family often take advantage of interactions outside the enzyme active site. We presently focus on peptide-based MMP inhibitors and probes that do not incorporate conventional Zn^2+^ binding groups. In some cases, these inhibitors and probes function by binding only secondary binding sites (exosites), while others bind both exosites and the active site. A myriad of MMP mediated-activities beyond selective catalysis can be inhibited by peptides, particularly cell adhesion, proliferation, motility, and invasion. Selective MMP binding peptides comprise highly customizable, unique imaging agents. Areas of needed improvement for MMP targeting peptides include binding affinity and stability.

## 1. Introduction

**Figure 1:**
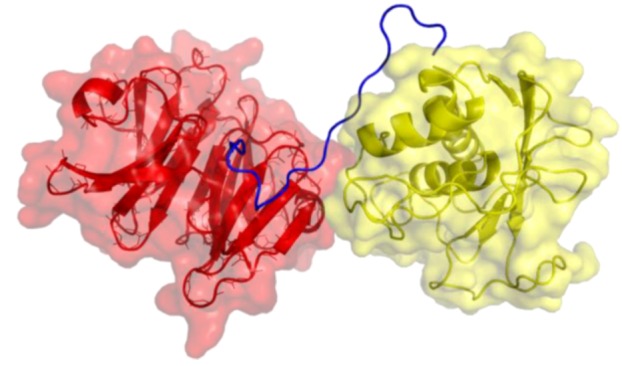
Three-dimensional structure of MMP-1. The catalytic (CAT) domain is yellow, the linker/hinge is blue, and the hemopexin-like (HPX) domain is red. The structure is based on X-ray crystallographic analyses of full-length MMP-1 [5,6].

**Figure 2:**
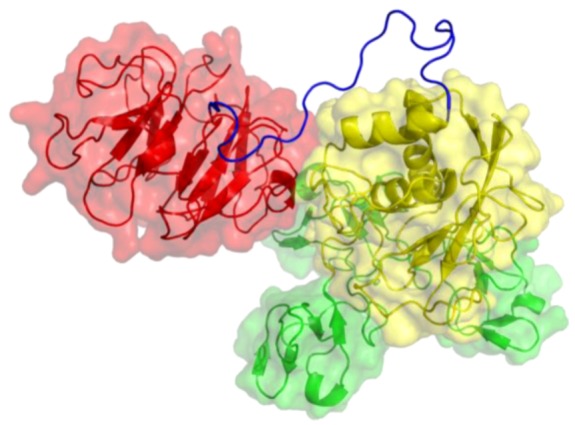
Three-dimensional structure of MMP-2. The catalytic (CAT) domain is yellow, the linker/hinge is blue, the hemopexin-like (HPX) domain is red, and the fibronectin type II [FN(II)] inserts are green. The structure is based on X-ray crystallographic analysis of full-length MMP-2 [7].

**Figure 3:**
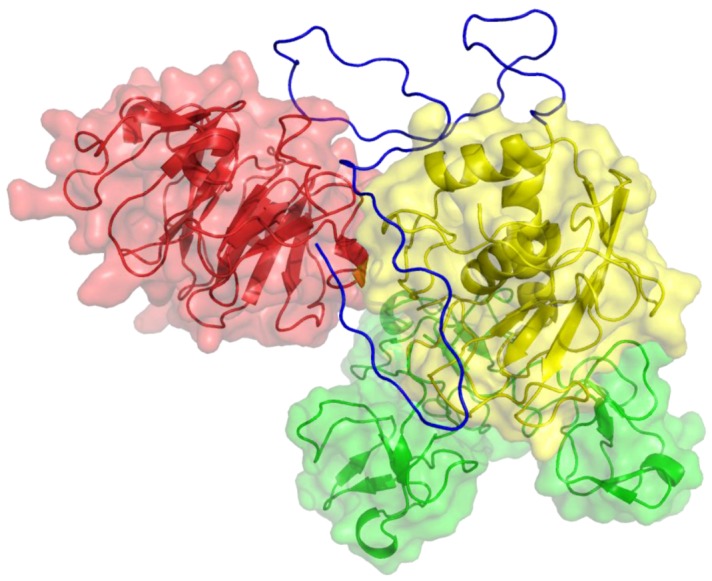
Three-dimensional structure of MMP-9. The catalytic (CAT) domain is yellow, the linker/hinge is blue, the hemopexin-like (HPX) domain is red, and the fibronectin type II [FN(II)] inserts are green. The structure is based on X-ray crystallographic analyses of MMP-9 CAT and HPX domains [8,9].

**Figure 4:**
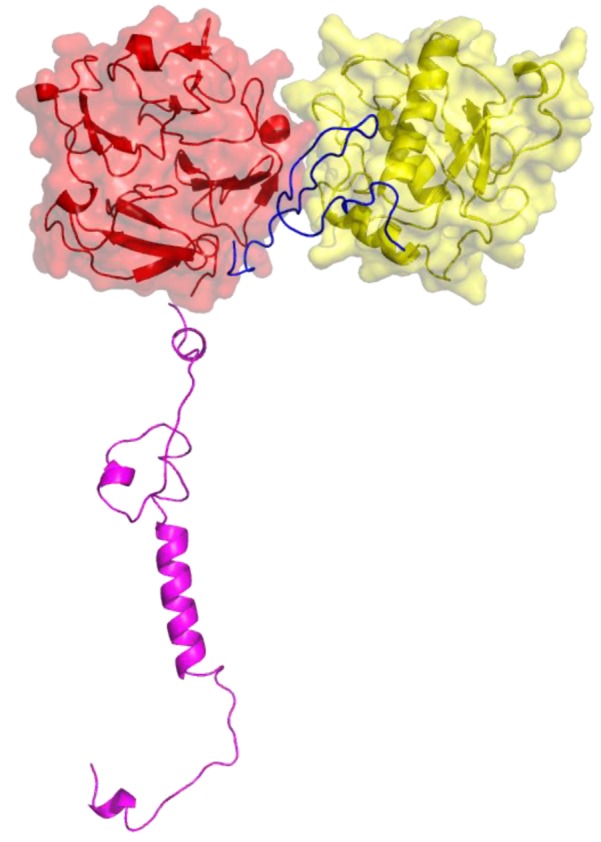
Three-dimensional structure of MT1-MMP. The catalytic (CAT) domain is yellow, the linker/hinge is blue, the hemopexin-like (HPX) domain is red, and the transmembrane and cytoplasmic domains are purple. The structure is based on X-ray crystallographic analyses of MT1-MMP CAT and HPX domains [10,11] and modeling of the transmembrane and cytoplasmic domains by I-TASSER [12,13].

Matrix metalloproteinases (MMPs) are part of a super family of zinc-dependent endopeptidases [1]. There are at least 23 human MMPs [2,3,4]. Most MMPs share a common domain organization, consisting of propeptide, catalytic (CAT), linker/hinge, and hemopexin-like (HPX) domains [2]. MMP-1 (Figure 1) is an example of this prototype. Two members of the MMP family possess three fibronectin type II [FN(II)] inserts in their CAT domain. These MMPs [MMP-2 (Figure 2) and MMP-9 (Figure 3)] are often referred to as “gelatinases” due to their initial identification as proteases capable of catalyzing the degradation of denatured collagen (gelatin). Several MMP family members have transmembrane domains that facilitate cell surface binding, and thus these MMPs are referred to as membrane-type MMPs (MT-MMPs). MMP-14/MT1-MMP (Figure 4) is representative of the MT-MMPs.

MMPs have long been recognized as potential targets for a variety of pathologies, including tumor angiogenesis and metastasis, osteoarthritis, inflammation, periodontitis, vascular diseases, post-myocardial infarction remodeling, neurodegenerative diseases, and neuropsychiatric disorders [14,15,16,17,18,19]. The development of MMP inhibitors has typically proceeded along the path of active site Zn^2+^ inhibition. The most common zinc-binding group used for this purpose is hydroxamic acid [20,21]. However, a primary reason hydroxamic acid-based inhibitors have not been successful in clinic trials is their lack of selectivity [21,22]. More recent work involves developing inhibitors that bind secondary binding sites (exosites) to pursue greater selectivity [23,24,25]. The present overview considers the use of peptides as MMP inhibitors and probes. Our discussion is restricted to peptides that interact with secondary binding sites of MMPs and/or exclude traditional zinc-interaction motifs.

## 2. Peptides That Target Gelatinase Members of the MMP Family (MMP-2 and MMP-9)

MMP-2 has been validated as an anticancer drug target in some aggressive tumors, while MMP-9 inhibition may have value in treating patients with early stage cancers [26]. MMP-9 has also been strongly implicated as a target for aberrant left ventricle remodeling post-myocardial infarction and multiple sclerosis progression [27,28]. Koivunen *et al.* used phage display peptide libraries to identify selective MMP-2 and MMP-9 inhibitors [29]. Peptide libraries such as CX_9_, CX_5-8_C, CX_3_CX_4_CX_2_C, *etc.* were displayed on filamentous phage and tested for their MMP inhibitory activity. From the screened and constructed peptides two cyclic sequences, CTTHWGFTLC (designated CTT; Table 1) and CRRHWGFEFC that contained a common HWGF motif, were found to be potent inhibitors of MMP-2 and MMP-9, respectively. The NMR-derived solution structure of CTT indicated a well-defined, saddle-shaped circular form [30]. In a gelatin degradation assay CTT inhibited MMP-2 with an IC_50_ value of 10 μM while CRRHWGFEFC preferentially inhibited MMP-9 relative to MMP-2 [29]. The latter peptide was not fully characterized due to solubility problems. Inhibitory effects were also studied for casein degradation, where CTT had an IC_50_ value of 5 μM for MMP-2 [29]. CTT did not inhibit MT1-MMP, MMP-8, or MMP-13 at concentrations up to 500 μM. 

**Table 1:** Peptide-based Inhibitors of MMP-Mediated Activities. ND = not determined.

Enzyme Affected	Peptide Sequence	IC_50_ (μM)	Reference
MMP-2	CTTHWGFTLC (CTT)	5–10	[29]
“	H-β^3^-Phe-β-Ala-β^3^-Trp-β^3^-His-OH	225	[31]
“	HWWQWPSSLQLRGGGS (M204C4)	78.0	[32]
“	HNWTRWLLHPDRGGGS (M205C4)	38.8	[32]
“	ISYGNDALMP (APP-IP)	0.030	[33,34]
“	MCMPCFTTDHQMARKCDDCCGGKGRGKCYGPQCLCR (Cltx)	0.200	[35]
“	CGAOGAOGSQGA (P713)	30	[36]
MMP-9	CRRHWGFEFC	ND	[29]
“	GACLRSGRGCG (TCTP-1)	ND	[37]
“	NQVDQVGY (IVS4)	50 ^a^	[38]
“	SRPQGPFL (IS4)	12 ^a^	[38]
“	FPGVPLDTHDVFQYREK (P3a)	109–279 ^a^	[39]
“	CQVTGALRSGRGKMLLC-NH_2_ (cyclic LRSG)	ND	[40]
“	CRVYGPYLLC	200 ^b^	[41]
	PRCBCGE (Regasepin1)	~1	[42,43]
“	RC-[D-B]-[D-R]	0.75	[44]
“	NENLLRFFVAPFPEVFG	50	[45]
“	CSCSDMTDKECLYFCMSEMS (STX-S4-CT)	1.0 ^d^	[46]
MT1-MMP	ISYGNDALMP (APP-IP)	2.0	[33,34]
“	GACFSIAHECGA (Peptide G)	150	[47]
“	AHQLH	165 ^c^	[48]
“	acetyl-VMDGYPMP-NH_2_ (IS4)	ND	[49]
“	acetyl-GYPKSALR-NH_2_ (IVS4)	ND	[49]
“	VFDEASLEP	238	Present study
MMP-1	CSCSDMTDKECLYFCMSEMS (STX-S4-CT)	4.5 ^d^	[46]

^a^ IC_50_ for cell migration; ^b^ IC_50_ for cell invasion; ^c^ IC_50_ for cell proliferation; ^d^ K_i_ value.

Using a transwell assay, CTT was shown to block migration of HT1080 fibrosarcoma, C8161 melanoma, SKOV-3 ovarian carcinoma, and KS1767 Kaposi sarcoma cell lines at significant levels in a dose dependent manner [29]. The cell migration of two endothelial cells (human Eahy926 and HUVEC) was also inhibited by the sequence. Control peptides did not affect cell migration. Due to the lack of a typical gelatinase substrate sequence, it was speculated that within the HWGF motif the Trp residue bound to the hydrophobic pocket of the enzyme while the His residue acted as a ligand for the catalytic zinc ion [29]. Complementing the CTT inhibitory properties, this sequence could be used to target tumors selectively as MMPs are over expressed in the tumor vasculature [29].

CTT has been utilized for radiopharmaceutical and imaging applications. ^125^I-[D-Tyr]-CTT exhibited an IC_50_ = 5–10 µM for MMP-2 [50]. Biodistribution studies in mice showed a tumor/blood ratio of 0.83 and tumor/organ ratios of 2.0, 2.56, and 6.6 for heart, muscle, and brain, respectively, at 120 min [50]. Large initial accumulation was observed in the liver and kidneys [50]. The modest tumor uptake and high concentrations in the liver and kidney was attributed to the high lipophilicity of ^125^I-[D-Tyr]-CTT. Ligand affinity may also have been diminished due to its monomeric nature, as opposed to the multivalent ligand presented in phage display. ^125^I-[D-Tyr]-CTT was 75% intact in serum after 10 min, with some release of ^125^I-D-Tyr [50]. Complete degradation occurred by 60 min. 

^125^I-Ala-Ala-Tyr-CTT and ^99m^Tc-CTT labeled CTT analogs have been prepared, where the first had the same MMP-2 inhibitory potency as the parent compound while the second was slightly less potent [30]. In mice bearing KS1767 Kaposi’s sarcoma xenografts, ^125^I-Ala-Ala-Tyr-CTT localized to the tumor, while ^99m^Tc-CTT labeled liposomes were used for tumor imaging [30]. CTT coated liposomes had enhanced tumor binding compared with unmodified liposomes, but the blood, kidney, and liver had high levels of label. 

^64^Cu-DOTA-CTT was found to inhibit MMP-2 (EC_50_ = 8.7 µM) and MMP-9 (EC_50_ = 18.2 µM) with similar affinity as CTT (EC_50_ = 13.2 and 11.0 µM, respectively), but *in vivo* was a poor tumor imaging agent due to its low affinity and instability [51]. ^111^In-DTPA-CTT (IC_50_ = 1026 µM for MMP-2) was found to accumulate in tumors possessing gelatinase activity, but offered low tumor contrast [52]. The stability of ^111^In-DTPA-CTT was ~85% in mouse serum after 3 h at 37 °C, so the affinity of the peptide appeared more problematic than the stability. Fusion of CTT with green fluorescent protein (GFP) allowed for fluorescence imaging of tongue carcinoma *in vivo* [53].

Incorporation of D-amino acids and certain secondary structural elements can help stabilize peptides against proteolytic degradation *in vivo*. A retro-inversion CTT, cyclic D-Cys-D-Leu-D-Thr-D-Phe-Gly-D-Trp-D-His-D-Thr-D-Thr-D-Cys, inhibited MMP-2 more efficiently than CTT [30]. Unfortunately, this peptide had limited water solubility. β-peptides have been shown to form stable secondary structures thereby emerging as a promising class of peptidomimetics [54,55,56,57]. The linear β-tetra peptide H-β^3^-Phe-β-Ala-β^3^-Trp-β^3^-His-OH was designed to mimic CTT. Mukai *et al.* found that H-β^3^-Phe-β-Ala-β^3^-Trp-β^3^-His-OH inhibited MMP-2 with an IC_50_ value of 225 µM, in comparison to CTT IC_50_ of 212 µM (as determined herein) [31]. Linear α-tetrapeptide had no significant contribution towards MMP inhibition, with an IC_50_ value of >1 mM [31]. While H-β^3^-Phe-β-Ala-β^3^-Trp-β^3^-His-OH has a similar affinity for MMP-2 as CTT, the new class of β-tetrapeptide is smaller in size and generally more stable, thereby enhancing its accumulation in target tissues.

Another peptide obtained from the above described phage display studies [29] was refined into an MMP-9 imaging agent [37]. Three modalities of the peptide TCTP-1 (GACLRSGRGCG) were produced; the linear form, a version with a disulfide bond between Cys3 and Cys10, and a version with a lactam. All three peptides had PEG(3)-DOTA added to the *C*-terminus and were labeled with ^68^Ga. The disulfide-bonded and lactam forms of TCTP-1 showed reasonable plasma stability, whereas the linear TCTP-1 was rapidly degraded. In a human melanoma xenograft model in rats, the disulfide-bonded TCTP-1 exhibited better tumor uptake than lactam TCTP-1. PET imaging demonstrated TCTP-1 uptake in the tumor, heart, liver, kidney, and urinary bladder. Overall, TCTP-1 tumor uptake was low, with weak correlation to MMP-9 expression in melanoma tumors.

A random 12-mer peptide phage display library composed of ~2.7 × 10^9^ sequences was screened for binding to full-length MMP-2 [32]. Twenty two clones were isolated as binders, and synthetic peptides corresponding to the binding sequences were synthesized and tested as MMP-2 inhibitors. Peptides M204C4 (HWWQWPSSLQLRGGGS) and M205C4 (HNWTRWLLHPDRGGGS) were found to inhibit MMP-2 hydrolysis of Mca-Pro-Leu-Gly-Leu-Dpa-Ala-Arg-NH_2_ with IC_50_ values of 78.0 and 38.8 nM, respectively. Both peptides inhibited pancreatic tumor cell invasion of Matrigel and reduced tumor growth in a human xenograft pancreatic tumor mouse model. However, neither peptide was stable in plasma after 10 min, requiring local use of the peptides for tumor treatment. Cyclic versions of each peptide were much less effective MMP-2 inhibitors.

Amyloid precursor protein (APP) is an integral membrane protein expressed in many tissues and concentrated in the neurons. After APP is proteolytically cleaved, a soluble extracellular domain containing an MMP-2 inhibitor is released and is believed to help protect the extracellular matrix from MMP-2 degradation [58,59,60,61]. The β-amyloid precursor protein-derived inhibitory peptide (APP-IP; sequence ISYGNDALMP), corresponding to residues 586–595 of APP770, is a highly selective MMP-2 inhibitor, where selectivity is based on unique structural features [33,34]. An IC_50_ value of 30 nM was observed for MMP-2, while MMP-9 and MT1-MMP IC_50_ values were in the μM range. Tyr588, Asp591, and Leu593 were shown to be important residues for optimal interaction between MMP-2 and the peptide. The APP-IP Asp6 carboxylate group coordinated the Zn^2+^ in the CAT domain of the enzyme; substitution with other residues leads to loss of inhibitory activity [34,62]. The inhibitor bound in the inverse direction as substrate.

Deshane *et al.* have shown that chlorotoxin (Cltx), a 36-amino acid peptide that was originally isolated from *Leiurus quinquestriatus* venom, inhibits the enzymatic activity of MMP-2 and causes a reduction in the surface expression of MMP-2 in glioma cells [63]. Immunohistochemical studies on Cltx revealed specific and selective binding of Cltx peptide to glioma cells but not to normal brain cells [64,65]. The remarkable specificity lead to the isolation and identification of a Cltx membrane receptor, which was MMP-2, co-purified as a major component of a stable macromolecular complex comprised of MMP-2, α_v_β_3_ integrin, MT1-MMP, and tissue inhibitor of metalloproteinase 2 (TIMP-2) [35]. A stoichiometric balance was required to facilitate tumor cell migration and invasion, where MT1-MMP activated MMP-2 and the α_v_β_3_ integrin promoted its maturation and release [66]. Western blotting analysis of Cltx treated with purified recombinant MMP-2, MMP-9, MMP-1, and MMP-3 resulted in only MMP-2 binding [35]. A dose-response curve was observed for MMP-2 inhibition, resulting in an IC_50_ of ~200 nM with complete inhibition in the presence of 300 nM Cltx. The Cltx binding site on MMP-2 is unknown. Cltx is a highly attractive therapeutic due to multiple tumors that express MMP-2, and the FDA has approved the use of Cltx in a Phase I/II clinical trial [65]. Cltx also has potential as an imaging agent; it been conjugated with Cy5.5 to allow imaging of glioma, medullolbastoma, prostate cancer, intestinal cancer, and sarcoma from adjacent non-neoplastic tissue in mouse models [67].

Unlike other MMPs, MMP-2 and MMP-9 have a collagen-binding domain (CBD) formed by three FN(II) modules. The CBD is the primary site of interaction for multiple collagens, gelatin, and elastin with MMP-2 and MMP-9 [68,69,70,71,72]. CBD-deleted mutants of MMP-2 had a 90% reduction in the rate of gelatin hydrolysis [73]. Shipley *et al.* demonstrated the inability of MMP-2 and MMP-9 to cleave elastin upon deletion of CBDs [70]. Using a combinatorial one peptide one bead library, Xu *et al.* determined that the CBD binds a short segment of the α1(I) collagen chain [36]. More specifically, CGAOGAOGSQGA (P713, where O = 4-hydroxyproline) was identified as an inhibitor of MMP-2 activity. P713 inhibited ~90% of MMP-2 gelatin cleavage (IC_50_ of ~30 µM), but less than 20% of the MMP-2 activity on a peptide substrate [NFF-1; Mca-Pro-Lys-Pro-Gln-Gln~Phe-Phe-Gly~Leu-Lys(Dnp)-Gly] which does not require the CBD for binding. To examine the specificity toward MMP-2, comparative inhibition assays were performed with MMP-8, with no alteration in MMP-8 activities observed upon P713 treatment [36].

Based on the single-stranded peptide model of the α1(I)715-721 collagen sequence identified above as a ligand for the MMP-2 FN(II) insert, our group assembled a triple-helical version of this ligand [α1(I)715-721 THP; (GPO)_4_-GAOGAOGSQGAO-(GPO)_3_-GPY-NH_2_] [74]. α1(I)715-721 THP inhibited MMP-2 and MMP-9 hydrolysis of α1(V)436-447 fTHP (Table 2), but did not inhibit MMP-2 or MMP-9 hydrolysis of a short, single-stranded substrate [Knight SSP; Mca-Lys-Pro-Leu-Gly-Leu-Lys(Dnp)-Ala-Arg-NH_2_] or a THP model of types I-III collagen (fTHP-15). To our knowledge, this demonstrated the first use of an exosite binder to selectively inhibit one collagen-based MMP activity (type V) but not another (types I–III).

**Table 2:** Inhibition of MMPs by α1(I)715-721 THP [74]. NI = no inhibition.

Enzyme	Substrate	K_i_^(app)^ (μM)
MMP-2	DQ gelatin	52.26 ± 5.110
“	Knight SSP ^a^	NI
“	fTHP-15 ^b^	NI
“	α1(V)436-447 fTHP ^c^	143.5 ± 11.40
MMP-9	DQ gelatin	54.42 ± 7.616
“	Knight SSP ^a^	NI
“	fTHP-15 ^b^	NI
“	α1(V)436-447 fTHP ^c^	122.7 ± 5.83

^a^ Knight SSP = Mca-Lys-Pro-Leu-Gly-Leu-Lys(Dnp)-Ala-Arg-NH_2_; ^b^ fTHP-15 = (Gly-Pro-Hyp)_5_-Gly-Pro-Lys(Mca)-Gly-Pro-Gln-Gly~Leu-Arg-Gly-Gln-Lys(Dnp)-Gly-Val-Arg-(Gly-Pro-Hyp)_5_-NH_2_; ^c^ α1(V)436-447 fTHP = (Gly-Pro-Hyp)_5_-Gly-Pro-Lys(Mca)-Gly-Pro-Pro-Gly~Val-Val-Gly-Glu-Lys(Dnp)-Gly-Glu-Gln-(Gly-Pro-Hyp)_5_-NH_2_.

Using confocal laser scanning and the multichannel character of existing DNA sequencers, Hu *et al.* screened several degenerate combinatorial peptide libraries and selected a novel MMP-9 inhibitor, regasepin1 (PRCBCGE, where B = biphenylalanine/Bip) [42,43,44]. Competitive enzyme inhibition assays using the substrate MIM-3b (POGPQGATGEOG), a fluorescent-labeled substrate, and gelatin, found regasepin1 inhibited MMP-9 in micromolar concentrations and in a dose-dependent manner. Regasepin1 was subsequently found to inhibit MMP-8, MMP-9, and ADAM17 equally well [42]. While the IC_50_ value for MMP-8 was 3 µM, MMP-1 and MMP-13 were inhibited with IC_50_ values of 100 µM. 3.1–7.5 mM regasepin1 protected mice against endotoxin shock [42].

The regasepin 1 backbone was used in a subsequent library that incorporated D-amino acids to develop relatively selective inhibitors of MMP-9 and ADAM17 [44]. The best inhibitor for MMP-9 was Arg-Cys-D-Bip-D-Arg (IC_50_ = 0.75 µM), while the best ADAM17 inhibitor was D-Pyr-D-Cys-Bip-D-Cys (IC_50_ = 0.6 µM). Modest selectivity was observed when comparing inhibitory potency between MMP-9, ADAM17, MMP-2, and MMP-3. For MMP-9, inhibition was by a mixed non-competitive mode.

Bacteria fermented milk has been shown to produce peptide inhibitors for proteases such as angiotensin converting enzyme (ACE), an enzyme involved in cardiovascular disorders [75,76]. Fermented milk is also thought to inhibit tumor growth directly or inhibit the proteolytic activity of tumor-associated stromal cells [77,78]. Juillerat-Jeanneret *et al.* evaluated peptides that had been described as ACE inhibitors for MMP inhibition. Two groups of peptides were synthesized and analyzed. These peptides incorporated the sequence -PFP- or -PLP- located at positions 42–44 of α_S1_-casein and 151–153 of β-casein, respectively. The peptide derived from α_S1_-casein inhibited MMP-2 and MMP-9 more efficiently than MMP-7. The most effective sequence for inhibiting MMP-9 from the -PFP-series was NENLLRFFVAPFPEVFG, with an IC_50_ value of 50 µM [45]. Peptides derived from β-casein had comparable IC_50_ values towards MMP-2, MMP-9, and MMP-7, with an increase in activity upon increasing the length of the *N*-terminus. The best inhibitor was VENLHLPLPLL, with IC_50_ values of 150, 250, and 300 µM for MMP-2, MMP-7, and MMP-9, respectively [45].

In most MMPs, the CAT domain is very similar, rendering it very difficult to construct selective inhibitors for each MMP. Diversity in the HPX domains among MMPs makes this exodomain a good target in the search for selective MMP inhibitors. The HPX domain is composed of four blades that consist of one α-helix and four antiparallel β-strands, giving them a propeller like structure. The MMP-9 X-ray crystallographic structure demonstrated that a homodimer is formed through blade IV of the HPX domain [9]. MMP-9 is presently thought to be the only secreted MMP that can form a homodimer, and the role of the homodimer is not well understood. Homodimer formation may be a prerequisite for MMP-9 induced cell migration, and therefore targeting homodimer formation was seen as a viable approach for inhibiting cell migration [79,80,81]. Using mammalian COS-1 cells, Dufour *et al.* tested the interaction interface of MMP-9 homodimer occurring through the outermost β-strand of the fourth blade of the HPX domain [38]. The outermost β-strand octapeptide (N688QVDQVGY695) was substituted with the corresponding region of the MMP-2 HPX domain to obtain a chimera MMP-9/IVS4. Furthermore, three additional mutants were made for the outermost β-strands of blade I, II, and III (MMP-9/IS4, MMP-9/IIS4, and MMP-9/IIIS4, respectively) to serve as controls. Using a co-immunoprecipitation assay, it was observed that mutants of blade I, II, and III had no effect on the homodimer formation while mutants of blade IV failed to dimerize. Evaluation of the four mutants in a transwell cell migration assay revealed mutant MMP-9/IVS4 failed to enhance cell migration in COS-1 cells in comparison to wild type MMP-9. Mutation of blade II and III had no effect on cell migration, while the blade I mutant MMP-9 failed to enhance cell migration. Inhibition assays for MMP-9 induced cell migration were carried out using NQVDQVGY (IVS4 peptide) and SRPQGPFL (IS4 peptide), designed to mimic the outermost β-strand of blade IV and blade I of the MMP-9 HPX domain, respectively. These peptides displayed a dose-dependent inhibition of MMP-9 induced cell migration, with IC_50_ values for IS4 and IVS4 of 12 and 50 µM, respectively. The IS4 peptide was found to disrupt interaction of MMP-9 with CD44.

The MMP-9 HPX domain was also found to bind to B chronic lymphocytic leukemia (B-CLL) cells via the α4β1 integrin [39]. Truncated variants of the MMP-9 HPX domain containing blades I and II (B1B2) or blades III and IV (B3B4) indicated that B3B4 interacted with the α4β1 integrin. Using peptides that span B3B4 (residues 621–707), peptide P3 from B4 (residues 654–674, PFPGVPLDTHDVFQYREKAYFC) inhibited cell adhesion to MMP-9. Asp660 and Asp663 mediated peptide P3 binding to the α4β1 integrin. A truncated version of peptide P3 (P3a, FPGVPLDTHDVFQYREK) bound to MEC-1 cells with a K_D_ = 282 µM. Peptide P3a also inhibited B-CLL and MEC-1 cell adhesion to proMMP-9 with IC_50_ = 279 and 109 µM, respectively. Peptide P3a inhibited B-CLL cell transendothelial migration and intracellular survival signals. 

Phage display analyses revealed the CRVYGPYLLC sequence as an MMP-9 HPX domain binder that inhibited homodimerization [41]. This peptide inhibited MMP-9 binding to the α5β1 and αvβ5 integrins in fibrosarcoma cells and inhibited fibrosarcoma cell invasion (50% inhibition at 200 μM peptide). The peptide did not inhibit MMP-9 catalyzed gelatin hydrolysis, and thus was a non-catalytic inhibitor [41]. Conversely, the cyclic LRSG peptide [CQVTGALRSGRGKMLLC-NH_2_, derived from MMP-9 HPX domain residues 615–622] inhibited MMP-9 interaction with the α5β1 integrin, tumor cell invasion, and cell surface gelatinolytic activity [40].

## 3. Inhibitors and Probes of the Membrane-Bound MMPs (MT1-MMP/MMP-14)

MT1-MMP is a critical protein in cancer invasion and metastasis [82]. MT1-MMP is up regulated in metastatic breast cancer patients in comparison to normal patients [83] and high expression of MT1-MMP has been correlated with low patient survival [84,85]. Using phage display libraries, the search for an MMP-2/MMP-9 inhibitor led to the preparation of a cyclic peptide with the sequence GACFSIAHECGA (Peptide G) as a control peptide due to its non-inhibitory properties [29]. Peptide G turned out to uniquely inhibit MT1-MMP. Peptide G inhibited MT1-MMP-mediated proteolysis of β-casein by 37% at 500 μM concentration. In the Quantizyme assay MT1-MMP inhibition by Peptide G was dose dependent with an IC_50_ value of 150 µM. The linear, scrambled peptide CGAAPEACGIHS had no effect on MT1-MMP activity. Peptide G had no inhibitory activity towards MMP-1, MMP-3, MMP-7, MMP-8, MMP-9, MMP-10, MMP-11, MMP-12, MMP-13, MMP-15, MMP-17, or MMP-20.

Peptide G also inhibited MT1-MMP-mediated proteolysis of the laminin Ln-332 γ2-chain by 64% at 100 µM. As the Ln-332 γ2-chain is hydrolyzed by a variety of carcinoma cells and linked to tumor progression [86,87], Peptide G inhibition of this process is an indication of its potential usefulness in the invasive phase of cancer. In transwell and matrigel assays, 100 µM Peptide G inhibited HT1080 fibrosarcoma and C8161 melanoma cell migration on fibronectin and invasion by 70 and 51%, respectively [47]. Peptide G inhibited soluble MT1-MMP activity in HSC-3 cell culture medium and increased the survival of mice bearing HSC-3 xenografts [47]. The potent nature of the peptide was attributed to its disulfide bond and restrained conformation. The full activity of peptide G was lost when the Cys residues were replaced by Ser or when Phe4 was altered. D-amino acid replacements led to a drop in activity, suggesting a strong conformational effect essential for efficient MT1-MMP binding.

Subtractive cell surface panning from phage random peptide libraries was used to identify peptides that targeted induced MT1-MMP and metal ions on MG-63 cells [88]. A prior study from this group using a phage dodecapeptide library and the CAT domain of MT1-MMP identified 13 binding peptides [48]. These peptides were combined with those obtained in the present study for a total of 35 peptides. Consensus sequences were AHQ/_S_LH/_P_, L/_I_/_E_PLL/_I_, T/Q/_D_ARH/_F_Q, and MK/_P_SR. Representative peptides AHQLH, LPLL, DTARFQ, and MKPSR docked with MT1-MMP at residues 120–125, where Zn^2+^ binding occurs. MG-63 and HepG2 tumor cell proliferation was examined in the presence of the representative peptides. All four peptides inhibited cell proliferation, with the maximum inhibition of 54% obtained with 100 µg/mL (~165 µM) AHQLH against MG63 cells.

MT1-MMP associates with CD44 and homodimerizes through its HPX domain, similarly to MMP-9. These associations lead to cell migration and invasion [89,90]. Detailed analysis of regions of the MT1-MMP HPX domain thought to promote cell migration and invasion led to the identification of sequences comprising the outer-strand of the individual HPX domain blades. Zarabbi *et al.* generated MT1-MMP chimeras by replacing the four outermost strands (S4) of the HPX domain with the corresponding regions of MMP-1 and examined the chimeras for their ability to induce cell migration [49]. Based on gelatin zymography, chimeras IS4 and IVS4 exhibited complete disruption of MT1-MMP activation of proMMP-2. From immunoprecipitation and Western blotting [38], the IVS4 chimera failed to bind wild-type MT1-MMP, suggesting homodimer formation required the outermost strand of blade IV. The same approach revealed that the interaction of CD44 with MT1-MMP required the outermost strand of blade I. A transwell chamber migration assay was performed to determine whether peptide sequences derived from MT1-MMP could interfere with MT1-MMP homodimer and CD44/MT1-MMP heterodimer formation. MT1-MMP mediated cell migration was significantly inhibited by the IS4 peptide acetyl-VMDGYPMP-NH_2_ and IVS4 peptide acetyl-GYPKSALR-NH_2_ while IIS4, IIIS4, and scrambled peptide had no effect on cell migration. IS4 and IVS4 were also found to inhibit metastasis *in vivo*. IS4 and IVS4 represented non-catalytic inhibitors, as catalytic inactivation of MT1-MMP had no effect on cell migration [91]. Interestingly, X-ray crystallographic analysis indicated that the MT1-MMP dimer interface involved interactions between blades II and III of one HPX domain with blades III and II of the other HPX domain [11]. Important residues for homodimerization were Asp385, Lys386, Thr412, Lys434, and Tyr436. The contrasting conclusions of the MT1-MMP dimerization studies, where interaction occurred between either HPX domain blades IV [49] or blades II and III [11], could have arisen from the different conditions that each study was performed under.

The outer blade regions of the MT1-MMP HPX domain have non-homologous loop sequences compared to other members of the MMP family. Our laboratory synthesized peptide models of the 5 MT1-MMP HPX domain loops (blade I strand 4, blade II strand 2, blade II strands 3–4, blade III strand 1, and blade IV strand 4), and examined their inhibitory activity for MT1-MMP processing of a triple-helical substrate (fTHP-17) (Table 3). Two peptides were micromolar inhibitors of MT1-MMP but did not inhibit MMP-1. VFDEASLEP-NH_2_, from blade II strand 3–4, had the best IC_50_ value, 238 µM. This peptide may directly or indirectly inhibit MT1-MMP dimerization (see above) or interaction of MT1-MMP with the substrate triple-helix. VRNNQV-[Nle]-DGYP-[Nle]-P-NH_2_, which is an *N*-terminal extension and modified version of the IVS4 peptide described above, was more effective than IVS4 for MT1-MMP inhibition of triple-helical peptidase activity (Table 3). When the complete cleavage reaction solutions were run over HPLC, peptides LFW-[Nle]-PNG-NH_2_ and RKDGKFV-NH_2_ both showed additional peaks indicating peptide processing by MT1-MMP.

**Table 3:** Inhibition of MMP Triple-helical Peptidase Activity by MT1-MMP HPX Domain Loop Peptides ^a^.

Enzyme	Loop Peptide Sequence	IC_50_ (μM) ^b^
MT1-MMP	LFW-[Nle]-PNG-NH_2_ (bIIIs1) ^c^	15,400
“	VFDEASLEP-NH_2_ (bIIs3-4)	240
“	RKDGKFV-NH_2_ (bIIs2)	3,000
“	VRNNQV-[Nle]-DGYP-[Nle]-P-NH_2_ (bIs4) ^c^	670
“	NNQKLKVEPGYPKSALRD-NH_2_ (bIVs4)	5,400
“	acetyl-VMDGYPMP-NH_2_ (IS4)	3,400
MMP-1	VFDEASLEP-NH_2_ (bIIs3-4)	NI
“	VRNNQV-[Nle]-DGYP-[Nle]-P-NH_2_ (bIs4) ^c^	NI

^a^ Concentrated peptide stocks were prepared, 80 µL of which was placed in a well of an opaque white 384 well plate, and serially diluted 1:2 nine times in triplicate. A buffer sample was included for 100% activity. Activated MT1-MMP or MMP-1 was added to each well at a concentration of 10 nM, shaken for 60 sec in a BioTEX plate reader, and incubated for 10 min at room temperature. 40 µL fTHP-17 [(Gly-Pro-Hyp)_4_-Gly-Pro-Lys(Mca)-Gly-Pro-Gln-Gly~Leu-Arg-Gly-Gln-Lys(Dnp)-Gly-Val-Arg-Gly-Leu-Hyp-Gly-Gln-Arg-Gly-Glu-Arg-(Gly-Pro-Hyp)_4_-NH_2_] was added to each reaction, the plate was shaken for 30 s in a BioTEK plate reader, and fluorescent readings were taken every 10 s for 10 min. Plates were sealed and placed in the dark at room temperature for a minimum of 24 h, and then read again for a fluorescent endpoint reading to facilitate enzyme activity calculation. Enzyme activity was calculated from the slope of the initial rate, and normalized as a percentage of the uninhibited MMP/fTHP-17 reaction. These normalized activities were plotted versus the log of micromolar inhibitor concentration in GraphPad Prism to determine approximate IC_50_ values for each peptide. 10 µL of each 250 µM reaction mixture was analyzed by C18 reversed-phase HPLC column to determine if the inhibitory peptide had been cleaved by MT1-MMP; ^b^ NI = no inhibition; ^c^ Met residues were replaced by norleucine (Nle).

An MT1-MMP near infrared probe was designed based on a peptide sequence identified in a phage display substrate library. The MT loop region of MT1-MMP is located within the CAT domain and contains an eight amino acid insertion unique to MT-MMPs (MT1-, 2-, 3-, and 5-MMP). To enhance selectivity, the phage display library was screened against this MT1-MMP sequence (^160^Arg-Glu-Val-Pro-Tyr-Ala-Tyr-Ile-Arg-Glu-Gly-His-Glu-Lys-Gln^174^, designated MT1-160p) rather than the complete sequence of MT1-MMP or its CAT domain. The non-substrate peptide MT1-AF7p (HWKHLHNTKTFL) displayed the highest affinity towards MT1-160p (K_D_ = 0.075 nM). MT1-AF7p bound via hydrogen bonding and hydrophobic interactions [92]. MT1-AF7p was labeled with Cy5.5 (Cy5.5-MT1-AF7p) and chosen for further validation *in vivo* [92]. The evaluation was performed in mice carrying MDA-MB-435 breast cancer xenografts (expressing high levels of MT1-MMP) or A549 xenografts (low MT1-MMP levels). MDA-MB-435 xenografts had significantly higher signal accumulation and better tumor contrast than the A549 xenografts. However, more precise quantitative data on tumor uptake and pharmacokinetics will be needed to determine the further utility of this probe.

## 4. Inhibitors of Matrix Metalloproteinase 1

MMP-1 is another MMP family member that has been validated as a cancer target [93]. TIMPs are well-characterized, high affinity, natural inhibitors of MMPs [94,95], and the TIMP scaffold has been utilized to pursue MMP-1 selective inhibitors. Modification of TIMPs can improve their selectivity within the MMP family [96,97,98]. Mini-TIMPs have been constructed using the sarafotoxin 6b fold [46], with the sarafotoxin 6b *C*-terminal residues VIW deleted to remove vasopressive activity. Substitutions were incorporated to make the sarafotoxin 6b sequence more “TIMP-like.” The best inhibitor was STX-S4-CT (CSCSDMTDKECLYFCMSEMS), which inhibited MMP-1 and MMP-9 with K_i_ = 4.5 and 1.0 μM, respectively. STX-S4-CT offered intriguing selectivity, as it did not inhibit MMP-3, MT1-MMP, or ADAM17. Binding simulations indicated complex interactions; both active site and exosite binding of STX-S4-CT with MMP-1 in ways reminiscent of TIMP binding and ways unique to STX-S4-CT. 

In related work from the same laboratory, phage display was utilized with TIMP-2 to identify selective inhibitors of MMP-1 [99]. Three regions of TIMP-2 underwent randomization, residues 2–6 (excluding residue 3), 34–40, and 67–70. TM8, which possessed Ser2 to Asp and Ser4 to Ala substitutions, offered the best selectivity. TM8 inhibited MMP-1, MMP-8, MMP-9, and MMP-13 with K_i_ values of 10, 28, 1.5, and 12 nM, respectively, while poorly inhibiting MMP-2, MMP-3, MMP-7, and MT1-MMP. Wild-type TIMP-2 effectively inhibits all of the aforementioned enzymes. These results reveal great potential for the design of MMP-selective mini-TIMPs.

## 5. Conclusions

MMP peptide-based approaches have yielded intriguing probes, including inhibitors that function on non-catalytic activities while leaving catalytic activities unaffected. For example, peptides based on MMP-9 and MT1-MMP HPX domain blade I strand 4 inhibit enzyme binding to CD44 while peptides based on blade IV strand 4 inhibit enzyme homodimerization. In turn, a peptide based on HPX domain blade II strands 3–4 inhibits MT1-MMP triple-helical peptidase activity. The main drawbacks of these probes have been their affinity and/or stability. In general, cyclization improves peptide stability, while sometimes offering improved affinity. Recent advances in phage display utilized mRNA to identify cyclic peptide inhibitors of thrombin with low nM affinities [100]. mRNA-display technology is further enhanced by incorporation of 12 unnatural amino acids. Thus, recent display technologies, in combination with methods for producing modified peptides, holds great promise for the future development of selective MMP inhibitors.
